# Expert Consensus Document: An Algorithm for the Care and Treatment of Patients with Constipation Based on Ultrasonographic Findings in the Rectum

**DOI:** 10.3390/diagnostics14141510

**Published:** 2024-07-12

**Authors:** Takaomi Kessoku, Masaru Matsumoto, Noboru Misawa, Momoko Tsuda, Yuka Miura, Ayaka Uchida, Yuki Toriumi, Tomoyuki Onodera, Hiromi Arima, Atsuo Kawamoto, Junko Sugama, Makoto Matsushima, Mototsugu Kato, Noriaki Manabe, Nao Tamai, Hiromi Sanada, Atsushi Nakajima

**Affiliations:** 1 Department of Palliative Medicine and Gastroenterology, International University Health and Welfare Narita Hospital, 852, Hatakeda, Narita 286-0124, Japan; kessoku-tho@umin.ac.jp; 2 Department of Gastroenterology, International University Health and Welfare Graduate School of Medicine, 4-3, Kozunomori, Narita 286-0048, Japan; 3 Department of Gastroenterology and Hepatology, Yokohama City University Graduate School of Medicine, 3-9 Fukuura, Kanazawa-ku, Yokohama 236-0004, Japan; nobomisa@yokohama-cu.ac.jp; 4 Department of Internal Medicine, Yokohama Clinic, Kanagawa Dental University, 3-31-6 Tsuruya-cho, Kanagawa, Yokohama 221-0835, Japan; 5 School of Nursing, Ishikawa Prefectural Nursing University, 1-1 Gakuendai, Kahoku 929-1210, Japan; matumoto@ishikawa-nu.ac.jp (M.M.); sanadah@ishikawa-nu.ac.jp (H.S.); 6 Department of Gastroenterology, National Hospital Organization Hakodate National Hospital, 18-16, Kawahara-cho, Hakodate 041-8512, Japan; momoko0221tsuda@gmail.com; 7 Department of Gastroenterology, Sapporo Cancer Screening Center, Public Interest Foundation Hokkaido Cancer Society, 1-15, Kita-26 Higashi-14, Higashi-ku, Sapporo 065-0026, Japan; mkato1957@gmail.com; 8 Research Center for Implementation Nursing Science Initiative, Fujita Health University, Aichi 470-1192, Japan; yuka.miura@fujita-hu.ac.jp (Y.M.); junko.sugama@fujita-hu.ac.jp (J.S.); 9 Department of Laboratory, Yokohama Clinic, Kanagawa Dental University, 3-31-6 Tsuruya-cho, Yokohama 221-0835, Japan; ayaka672028@gmail.com (A.U.); yukikotoriumi0626@gmail.com (Y.T.); 10 Department of Clinical Laboratory, National Hospital Organization Hakodate National Hospital, Hokkaido 041-8512, Japan; onodera.tomoyuki.rx@mail.hosp.go.jp; 11 Department of Radiological Technology, Coloproctology Center Takano Hospital, Kumamoto 862-0971, Japan; h11arima@yahoo.co.jp; 12 Division of Ultrasound and Department of Diagnostic Imaging, Tokyo Medical University Hospital, 6-7-1 Nishishinjuku, Shinjuku-ku, Tokyo 160-0023, Japan; a-kawamo@tokyo-med.ac.jp; 13 Matsushima Hospital Proctology Center, 9-11, Honcho, Tobe, Nishi-Ward, Yokohama 220-0041, Japan; makotomd@matsushima-hp.or.jp; 14 Division of Endoscopy and Ultrasonography, Department of Clinical Pathology and Laboratory Medicine, Kawasaki Medical School General Medical Center, Okayama 700-8505, Japan; n_manabe@med.kawasaki-m.ac.jp; 15 Department of Nursing, Graduate School of Medicine, Yokohama City University, Yokohama 236-0027, Japan; tamai.nao.tx@yokohama-cu.ac.jp

**Keywords:** constipation, fecal retention, rectum, ultrasonography, treatment, defecation care

## Abstract

Chronic constipation is a common gastrointestinal disorder, and its management is critical. However, it is extremely difficult to assess its subjective symptoms when patients are unable to report them due to cognitive or physical disabilities, especially in cases of patients with incurable geriatric, pediatric, palliative, psychiatric, or neurological diseases. We had previously established a protocol for observing and assessing rectal fecal retention using ultrasonography and for classifying cases into three categories based on the rectal findings: no fecal retention, fecal retention without hard stools, and fecal retention with hard stools. However, although the detection of rectal fecal retention using ultrasonography would be expected to lead to better therapeutic management, there is no standard algorithm for selecting specific treatments and defecation care options based on ultrasonographic findings. Therefore, we organized an expert consensus meeting of multidisciplinary professionals to develop such an algorithm based on rectal ultrasonography findings for patients with constipation in both residential and hospital settings.

## 1. Introduction

Chronic constipation is a common gastrointestinal disorder that affects over 16% of adults worldwide. Its incidence is higher among older individuals—approximately 33% of adults aged 60 years or older report at least occasional constipation, and the prevalence is 50% or more among nursing home residents [1,2]—as well as among women and individuals in lower socioeconomic status groups [3,4]. Recently, the prognosis of patients with cardiovascular conditions and constipation has been shown to be significantly worse than that of patients without constipation, with the former being at an increased risk of cardiovascular events, possibly due to, at least in part, increased cardiovascular stress during defecation [5]. Moreover, an epidemiological study in Japan reported a significant increase in cardiovascular events with a decrease in the frequency of defecation [6]. Therefore, the management of chronic constipation is crucial.

The Rome IV criteria categorize chronic constipation disorders into four types: (a) functional constipation, (b) irritable bowel syndrome with constipation, (c) opioid-induced constipation (OIC), and (d) functional defecation disorders, including inadequate defecatory propulsion and dyssynergic defecation [7]. Functional constipation is the most common type of chronic constipation and is generally diagnosed based on symptoms according to the Rome IV diagnostic criteria [1]. For a diagnosis of functional constipation, two or more of the following symptoms should be present: straining during defecation, lumpy or hard stools, sensations of incomplete evacuation, sensation of anorectal obstruction or blockage, need for manual maneuvers, and greater than three bowel movements per week. However, three of the six Rome IV items are subjective, and their evaluation is extremely difficult if patients are unable to communicate because of cognitive or physical impairments. Hence, it is necessary to assess colonic fecal retention in patients using more objective methods.

The diagnostic procedures typically recommended for evaluating the rectum and colon for constipation include plain abdominal radiography, barium enema, colonoscopy, defecography, abdominal computed tomography (CT), and magnetic resonance imaging [8,9,10]. However, these procedures have certain disadvantages, such as invasiveness, radiation exposure, long examination times, and not providing adequate information. Moreover, they are relatively expensive, unsuitable for follow-up testing, and lack standardization. Radiography can be performed at many hospitals, but the cost is moderate, and the findings are often unclear. Conversely, conventional ultrasonography can be broadly applied in clinical practice because of the advantages of being quick, cost-effective, non-invasive, safe, and involving non-ionizing radiation [11,12]. Recently, there has been a dramatic increase in the use of handheld ultrasound devices for point-of-care ultrasound (POCUS) by physicians and nurses who do not specialize in ultrasonography. Such handheld devices enable them to examine patients at the bedside and make on-the-spot decisions related to patient care. Ideally, POCUS can be used by any member of a multidisciplinary team to assess whether fecal retention is causing constipation. Therefore, the use of ultrasonography in the treatment of constipation has the following advantages: (1) radiation-free, (2) simple and quick, (3) repeatable, (4) inexpensive compared to CT or MRI, and (5) It can be used not only in hospitals but also at home and in nursing care settings.

Ultrasonographic detection of colonic fecal retention has been shown to be useful for diagnosing constipation and evaluating treatment effectiveness. Manabe et al. have reported that the responsiveness of patients with chronic constipation to medical treatment depends on parameters such as the constipation index and left/right distribution ratio, both of which are used to evaluate stool and/or gas distribution and are calculated using ultrasonographic observations of the colon. This suggests that the entire colon should be examined using ultrasonography for a proper assessment of constipation. However, all members of a multidisciplinary team may not be experienced enough for this type of assessment. The currently available evidence emphasizes the importance of determining the presence or absence of rectal fecal retention when assessing constipation in older adults; additionally, several educational programs related to ultrasonography have recently been developed for nurses, and the effectiveness of rectal ultrasonography-based defecation care in a home-care setting has been verified. 

To build on this previous work, we developed an observation protocol through expert consensus [13] and created a flowchart that can be used to confirm the presence, properties, location, and volume of rectal fecal retention in cases of suspected constipation [13]. Transverse transabdominal approach ultrasonographic images are used to confirm the presence or absence of rectal fecal retention and categorize each case as being one of stool retention, hard stool retention, or no stool retention. We also demonstrated that ultrasonography findings were highly consistent with CT findings of fecal mass retention in the rectum. Nonetheless, although this flowchart allows physicians, laboratory technicians, and nurses to evaluate fecal retention using rectal ultrasonography, it does not have any provision for indicating which treatment or defecation care methods should be selected based on the findings. Currently, guidelines on constipation state that the presence or absence of rectal fecal impaction can be assessed using echocardiography [13,14,15], but have not yet clearly indicated care or treatment based on that classification, but this is the first time that a consensus on care and treatment algorithms based on rectal ultrasonographic findings has been developed. There are three main reasons for this: (1) cost-effectiveness (reducing unnecessary disimpaction, suppository and enemas), (2) assurance of patient safety, and (3) improvement of patient satisfaction. Currently, transanal procedures such as disimpaction, suppositories, and enemas are performed blindly. However, if there is no stool in the rectum, these care and treatment approaches are ineffective and, therefore, are futile medical procedures. Furthermore, it is to the patient’s detriment to perform transanal procedures even when there is no stool in the rectum, as this can lead to complications such as perforation of the gastrointestinal tract or hemolysis. Therefore, we sought to develop an algorithm for selecting treatments and defecation care methods based on rectal ultrasonography findings.

## 2. Materials and Methods

### 2.1. Design

Our aim was to create an algorithm for selecting treatment and defecation care methodologies for suspected chronic constipation, especially rectal constipation, based on rectal ultrasonography findings. This consensus statement is intended to serve as a guide for physicians and nurses working in the hospital and home care settings who do not specialize in ultrasonography or the diagnosis of chronic constipation. For a narrative review of the current literature in this area, we convened a group of gastroenterologists, proctological surgeons, ultrasound specialists, internal medicine experts, nurses, and a wound, ostomy, and continence nurse (WOCN) with expertise in ultrasonography and evaluation of chronic constipation. Group members were selected on the basis of their research achievements with regard to constipation evaluation and treatment or defecation care in Japan. Online working-group meetings were held once a month (at least 10 meetings in total) between January 2023 and October 2023 to develop the consensus statement content. Subsequently, another meeting was conducted so that all members could reach a consensus. 

This novel algorithm is based on our previous flowchart for evaluating rectal fecal retention using ultrasonography [13] and a practice algorithm used during colonic fecal impaction assessment in nursing care [14], with additional inputs based on expert opinions on treatment and defecation care according to specific ultrasonography findings. The content of this consensus document was presented at the third meeting of the study group for ultrasonography for chronic constipation on 28 October 2023 in Tokyo, Japan, and a final version was developed after further discussion and agreement. 

### 2.2. Rectal Ultrasonography Protocol

**Figure 1:**
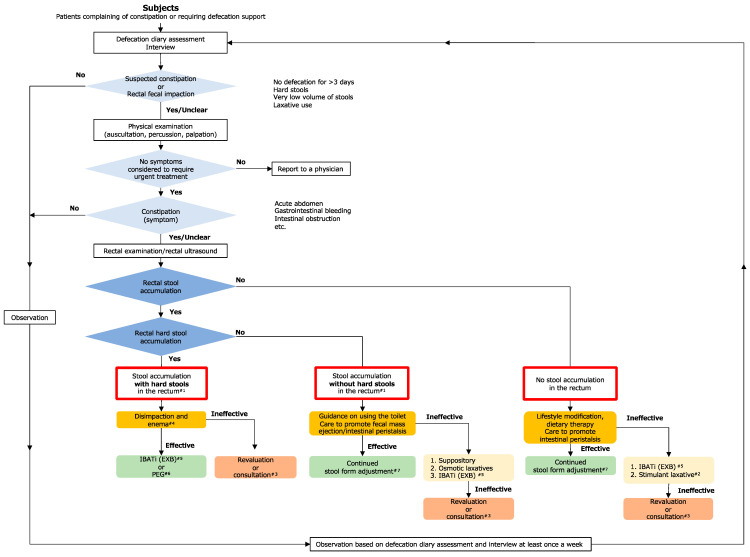
The flowchart of recommended care and treatments for rectal constipation based on ultrasonography findings. ^#1^ Multiple echoes, indicating gas storage. ^#2^ Exercise caution initially in the absence of urgent medical conditions, such as intestinal obstruction. ^#3^ Includes reporting and consultation from nurses to physicians and consultation from physicians to specialists. ^#4^ If the patient is able to expel fecal masses on his/her own, encourage self-defecation first. ^#5^ In the absence of the desire for defecation, the use of an IBATi (e.g., EXB) is preferred. ^#6^ Refrain from using stimulant laxatives, considering the risk of intestinal perforation. ^#7^ If the Bristol Stool Form scale classification is 1–2, use an osmotic laxative (MgO or PEG). EXB; elobixibat, IBATi: ileal bile acid transporter inhibitor, MgO; magnesium oxide, PEG; polyethylene glycol.

The target population for the ultrasonographic observational protocol comprises individuals with suspected constipation. In accordance with our previous reports [13,14], rectal ultrasonography was performed irrespective of whether patients could or could not complain of constipation (Figure 1). Subjects who could be interviewed were evaluated for suspected constipation according to the Rome IV diagnostic criteria [7]. Objective information must be obtained from participants who cannot be interviewed—for example, the Bristol Stool Form scale should be used to assess fecal characteristics, and constipation should be suspected when hard, category 1 or 2 stools are observed. Constipation should also be suspected when the stool volume is clearly low, considering food intake. It is preferable for ultrasonography to be performed when there is urine retention in the bladder (since the bladder can then be used as an acoustic window) when the patient has not eaten immediately prior to the examination (since there is no effect of small bowel contents or digestive gases), and when there is no gas retention in the abdomen.

### 2.3. Methods and Classification

**Figure 2:**
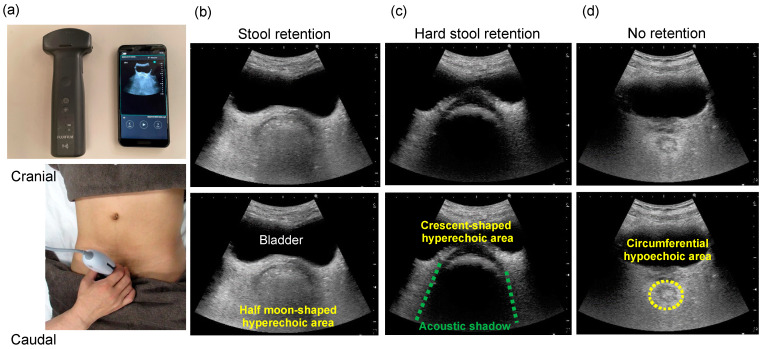
Transverse rectal ultrasound images showing the presence of stools and hard stools. The top three images are original ultrasound images, and the bottom three images illustrate the ultrasound findings. (**a**) Echo probe application procedure using the wireless iViz air^®^ (Fujifilm, Tokyo, Japan) ultrasonography device. The probe is placed at the superior margin of the pubis for transverse scanning. The ultrasound beam is tilted 10–30 degrees caudally to visualize the bladder, which is used as an acoustic window, and the rectum is visualized deeper than the bladder. (**b**) Stool retention. A half-moon-shaped hyperechoic area is observed in the lower part of the bladder. (**c**) Hard stool retention. A crescent-shaped hyperechoic area with an acoustic shadow is observed in the lower part of the bladder. (**d**) No retention. No hyperechoic area is observed because there is no fecal retention. A circumferential hypoechoic area is observed in the lower part of the bladder.

According to our previous protocol [13,14], the subject was placed in the supine position, and the probe was positioned at the suprapubic border. The detection of a semilunar or crescent-shaped high-echo area located dorsal to the bladder in a transverse scan is considered indicative of fecal mass accumulation in the rectum. If a hard fecal mass is present, both a clear crescent-shaped echogenic region and an acoustic shadow are observed. If the diameter of the crescent-shaped, strongly hyperechoic area is ≥4.5 cm, a fecal embolus is suspected; in this condition, a large, hard stool occupies the rectum and cannot pass through the anus by itself. The final conclusion of using this transverse-applied ultrasound to the rectum can be classified into three categories: no fecal retention, fecal retention without hard stools, and fecal retention with hard stools [13] (Figure 2).

A transabdominal approach is generally utilized; however, it may not be practical in case of an empty urinary bladder or a lot of gastrointestinal gas. In such cases, the transgluteal approach can be used [16]. In this variation, the subject is placed in the supine position with the knees flexed, and the probe is placed over the gluteal cleft. As with the transabdominal approach, observation of a high-echo area is considered indicative of fecal retention. 

### 2.4. Assessment-Based Selection of Nursing Care Interventions

Assessment-based care includes care to promote stool mass evacuation or intestinal peristalsis and/or dietary and drug adjustments [14].

#### 2.4.1. Care to Promote Stool Mass Discharge

- Fecal disimpaction

Fecal disimpaction is performed in case the patient is unable to defecate spontaneously or apply abdominal pressure, for example, due to paralysis or rectal–anal dysfunction [17]. It is especially indicated for patients with suspected fecal embolization and those with difficult-to-defecate constipation who fail to defecate even after the use of suppositories and enemas. As the stool extraction procedure can lead to complications such as bleeding due to damage to the rectal mucosa, rectal perforation, and hypotension due to the vagal reflex, the procedure should be performed with extreme caution.

First, lubricate the gloved fingers using a lubricant. Position the patient in the left lateral recumbent position, tap the anus with a finger, and insert the finger gently and slowly (6–8 cm) once the anus relaxes. Remove the stool clumps from the rectal wall and break up any large clumps before their removal.

- Enema, suppository

Position the patient in the left lateral recumbent position, insert a tube through the anus, and slowly inject a small amount of enema solution (up to 50 mL) warmed to approximately 40 °C. In cases of fecal embolization (large, hard stools blocking the anus), stretching of the intestinal tract and decreased blood flow due to the presence of the enema solution can result in bleeding or rectal perforation. Glycerin used in enemas promotes defecation by facilitating catharsis and increasing the contractility of the distal colon and rectum. However, its effectiveness decreases with increased tolerance due to repeated use, making long-term use undesirable [18]. In case of bleeding, the presence of glycerin in the blood can cause hemolysis, and, therefore, the procedure should be performed with caution. In addition, patients with cognitive impairment or age-related decline in external anal sphincter contractility may not be able to control their bowel movement after the procedure, leading to fecal incontinence.

Suppositories can be of two types: those that promote fecal evacuation by generating carbon dioxide in the rectum to increase intrarectal pressure and those that promote fecal evacuation by acting directly on the rectal mucosa to promote peristalsis.

- Biofeedback

This method effectively reinforces training by converting biological responses into light, sound, or other signals to obtain visual, auditory, or other detectable feedback of biological information. In practice, pelvic floor muscle dyscoordination can be improved by teaching patients how to apply abdominal pressure when leaning forward and straining during defecation and by making them aware of their anorectal movements using an anal electromyograph, anal manometry, or a rectal balloon. The “Evidence-based clinical practice guidelines for chronic constipation 2023” [15] describe the indication for biofeedback as a functional defecation disorder due to pelvic floor muscle incoordination disorder. This condition is characterized by the failure of the pelvic floor muscles, including the puborectalis and anal sphincter muscles, to relax properly during defecation.

The training method uses electromyography biofeedback training equipment and a medical electromyography system to generate an anal electromyograph. To ensure that the abdominal muscles are sufficiently contracted to increase the abdominal pressure while simultaneously keeping the pelvic floor muscles relaxed and uncontracted, or for their proper guidance, one channel for the abdominal muscles and another for the pelvic floor muscles is used to simultaneously display and record both surface electromyograms. When using an anal manometer, an anal pressure microtransducer is used; the sensor is inserted into the anus, and the actual movement of the anal sphincter is monitored during training. When a rectal balloon is used, training is performed by inserting it into the rectum and pushing it out like stool.

Biofeedback therapy has been reported to improve constipation symptoms by approximately 70% [19], and biofeedback therapy is effective in 71% of patients with pelvic floor dysfunction [20]. However, because it is a highly specialized treatment, it should be performed at a specialized facility [15].

- Pelvic floor muscle exercises

These involve pelvic floor muscle contraction training for the prevention and treatment of urinary and fecal incontinence and pelvic organ prolapse. Pelvic floor muscle relaxation training in biofeedback therapy for pelvic floor muscle incoordination may have a secondary effect, but there is a lack of evidence that it improves constipation.

- Forced defecation method

The goal of this method is periodic emptying of the colon, with an adequate amount of stool expelled at once to eliminate residual stool. Both retrograde and progressive ablutions can be performed. In retrograde colon irrigation, water injected through the anus is allowed to reach the cecum with the goal of enabling excretion of the contents of the entire colon at once. Forced defecation, which is currently covered by insurance, is also called “transanal self-irrigation” and is referred to medically as “transanal irrigation” or “retrograde colon irrigation”. It is a treatment for preventing fecal incontinence and improving constipation symptoms and involves injecting 300–1000 mL of slightly warm water into the rectum transanally once every 1–2 days to enable evacuation of as much of the contents of the rectum and the left-side colon as possible. Long-term retrograde colonic irrigation has been reported to be beneficial in 45% of patients with defecation disorders [21].

In Japan, the Peristine^®^ anal irrigation system was approved by the Japanese pharmaceutical affairs in 2016 for use in transanal bowel cleansing therapy. The reimbursement has been approved for calculation as the “home transanal self-bowel cleansing instruction and management fee” since 2018, and an additional fee for materials was added in 2021. The indication for this treatment is defecation disorders caused by spinal cord disorders that do not improve sufficiently after more than 3 months of conservative treatment. 

Antegrade continence enema is performed by connecting an abdominal inlet to the colon and injecting an enema solution into the ascending colon to excrete feces from the entire colon at once. The appendix is surgically separated from the cecum, that part is sutured, the appendix is reversed, and the distal side is sutured through the submucosal tunnel of the cecum. The appendage is then placed in an anti-reflux mechanism, and one end of the appendage is placed as an inlet into a hole in the abdomen. Through this narrow passage, the colon can be accessed via a catheter. Pediatric urologists and surgeons sometimes perform these procedures on patients with spina bifida who require treatment during childhood.

#### 2.4.2. Care to Promote Intestinal Peristalsis

- Lifestyle improvements

The “Evidence-based clinical practice guidelines for chronic constipation 2023” recommend “appropriate diet, exercise, and abdominal wall massage” for improving symptoms of chronic constipation [15]. Exercise therapy, especially aerobic exercise, has been reported to be effective in improving chronic constipation symptoms [22]. Abdominal wall massage for 15 min a day, 5 times a week, has also been reported to be effective in relieving chronic constipation [23,24]. 

#### 2.4.3. Dietary and Medication Adjustments

- Dietary treatment

Diets should include foods that soften the stool and stimulate bowel movements, including fermented foods and foods rich in soluble and insoluble fiber. Supplements should be prescribed to patients with chewing or swallowing problems and those who cannot consume sufficient amounts of the recommended foods.

- Probiotics

Probiotics are defined as “live microorganisms that have a beneficial effect on the health of the host when consumed in appropriate amounts”. The beneficial effects of probiotics are attributed to improved hemostasis of the intestinal microflora. According to the guidelines [15], “certain probiotics are effective in increasing the frequency of defecation and improving abdominal symptoms in patients with chronic constipation”.

- Pharmacotherapy using laxatives

The type, mechanism of action, and generic and proprietary names of various laxative drugs are summarized in Table 1. 

Conventional laxatives include osmotics and stimulants; in recent years, intestinal secretagogues, ileal bile acid transporter (IBAT) inhibitors, and osmotic laxatives have emerged as newer alternatives.

Regarding the mechanisms of action, drugs for constipation can be divided into four categories based on whether they induce intestinal water secretion, stimulate the large intestine, recover the loss of defecation desire (LODD), or block OIC (Figure 1). If there is a possibility of OIC, which is defined as treatable drug-induced constipation according to the latest guidelines, the administration of naldemedine—the only approved drug for OIC—should be initiated first. Currently, the constipation symptoms associated with weak opioids are not well recognized, and because the proportions of patients with constipation due to exposure to weak and strong opioids are similar [25], it is recommended that naldemedine be co-administered with all opioids [26]. Constipation after naldemedine therapy can be diagnosed as constipation other than OIC, thus simplifying the diagnosis. Naldemedine is an OIC-specific drug; for constipation other than OIC, it is important to understand the characteristics of various osmotic laxatives, epithelial function-altering agents, bile acid reuptake inhibitors, and topical agents (Table 1). 

**Figure 3:**
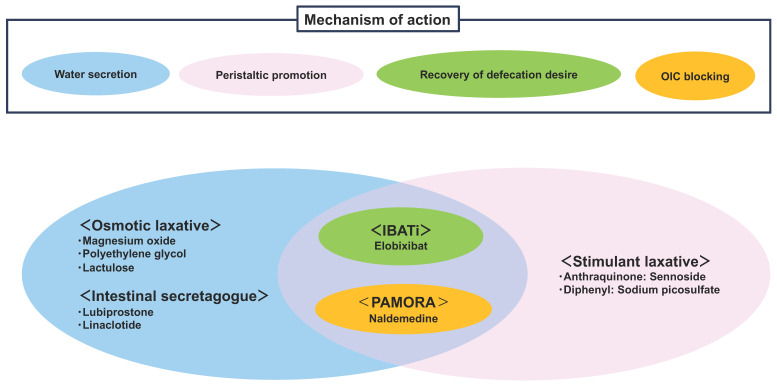
Positioning of each constipation drug according to the mechanism of action. OIC: opioid-induced constipation, IBATi: ileal bile acid transporter inhibitor, PAMORA: peripherally acting μ-opioid receptor antagonist.

The first step in drug selection is to be aware of the risks of hypermagnesemia and of factors that can reduce the effect of magnesium [26]. Understanding their mechanisms of action is also critical. Osmotic laxatives and intestinal secretagogues induce intestinal water secretion, whereas stimulant laxatives promote colonic peristalsis. Elobixibat is an agent that combines all three actions (water secretion, promoting colonic peristalsis, and recovery of LODD) by inhibiting bile acid reabsorption in the terminal ileum and increasing bile acid influx into the colon (Figure 3). 

Finally, it is also important to consider the burden of nursing and caregivers. For example, preprandial medications increase the burden on nurses, caregivers, and family members because of their increased distribution, due to which extra care must be taken in terms of adherence and proper use. Another notable point is that the powdered formulation of polyethylene glycol must be dissolved in water, which requires caution in patients who have difficulty swallowing or take a long time to ingest fluids. It is very important to consider such cautionary factors, the various mechanisms of action of the drugs, and the nursing and care burden as well as the situation of the patient, medical staff, and others involved when selecting the most appropriate drugs to be used [26].

1. Osmotic laxatives
  (i) Magnesium oxide
    Osmotic laxatives increase defecation frequency by inducing intestinal water secretion. Magnesium oxide (MgO) is a common osmotic laxative, and although it is inexpensive and its long-term administration has been reported to be safe, electrolyte abnormalities can cause dehydration and bradycardia, and overdoses can, therefore, be especially problematic in patients with renal and cardiac failure [27]. Oral MgO administration has been reported to increase serum magnesium levels in patients with renal failure [28], and the “Guidelines for Safe Pharmacotherapy of the Elderly 2015” published by the Japanese Geriatrics Society recommend that older patients with renal dysfunction should not receive MgO because of the increased risk of hypermagnesemia [29]. The reported risk factors for hypermagnesemia include age ≥ 68 years, renal dysfunction, MgO dosage > 1650 mg/day, and administration of MgO for >36 days [30]. Additionally, as gastric juices convert MgO into magnesium salts, their effectiveness is generally reduced to less than 50% in patients who have undergone total gastrectomy or are taking acid-secretion inhibitors [31]. When MgO is administered in daily practice, care should be taken regarding surgical and medical histories, especially the administration of proton pump inhibitors and histamine 2 blockers. Caution should also be exercised with regard to the concomitant administration of medications such as acid secretion inhibitors, bisphosphonates, non-steroidal anti-inflammatory drugs, and antiepileptic drugs, as it reduces their efficacy by inhibiting their absorption. Thus, although MgO is widely used, it should be used with caution and after ensuring that renal function will not be impaired.
  (ii) Polyethylene glycol
    The osmotic effect of polyethylene glycol promotes water secretion into the intestinal tract and exerts a laxative effect [32].
  (iii) Lactulose
    Lactulose reaches the lower gastrointestinal tract without being digested or absorbed, increases the osmotic pressure in the intestine to promote water secretion, and is metabolized by intestinal bacteria to produce organic acids that increase intestinal peristalsis and have a laxative effect. It was reported to be significantly effective in a randomized, double-blind, placebo-controlled study in patients with chronic constipation in Japan [33].
2. Stimulant laxatives
  Stimulant laxatives include anthraquinones such as sennosides and aloe, and diphenyls such as sodium picosulfate. Both are hydrolyzed to their active forms by intestinal bacteria and enzymes in the digestive tract, which then act on the intermuscular plexus of the colon to promote high-amplitude propagated contractions (HAPCs), inhibit water absorption from the intestinal tract, and produce purgative effects [25,34]. Anthraquinone-based stimulant laxatives are widely used in Japan; however, no randomized controlled trials have investigated their efficacy in the treatment of chronic constipation. In contrast, the diphenyls sodium picosulfate and bisacodyl have been shown to be effective [35,36,37]. As anthraquinones can cause intractable constipation owing to the emergence of tolerance after long-term continuous use, they should be used only when necessary, under supervision, and for short durations or rescue use [15].
3. Intestinal secretagogues
  (i) Lubiprostone activates the CIC-2 chloride channel on the luminal side of the small intestine and stimulates osmotic secretion of water into the intestinal tract, softening stool and promoting defecation [38]; side effects include nausea and diarrhea. It has been shown to significantly improve the symptoms of OIC and was well tolerated by patients with chronic non-cancer pain [39]. Another advantage is its capsule-based formulation; it is administered once or twice daily after meals and reduces the burden on nurses and caregivers.
  (ii) Linaclotide improves gastrointestinal tract hypersensitivity by increasing cyclic guanosine monophosphate levels in intestinal epithelial cells and promoting intestinal fluid secretion and defecation. It has been shown to be effective in treating constipation-type irritable bowel syndrome [40].
4. IBAT inhibitors
  Elobixibat inhibits IBAT expressed on epithelial cells in the terminal portion of the ileum, increasing the amount of bile acid entering the colon, which in turn increases water and electrolyte secretion into the intestinal tract, enhances intestinal peristalsis and promotes defecation [41]. Regarding constipation in cancer patients, an increase in the frequency of spontaneous bowel movements has also been observed, and elobixibat treatment has been shown to be unaffected by the amount of food consumed. Therefore, it is suggested that elobixibat may be used by cancer patients regardless of dietary intake [42]. The decrease in rectal sensory thresholds due to the increase in bile acid levels has restorative effects on the desire to defecate [43,44]. Thus, besides promoting water secretion and intestinal peristalsis, it also recovers the LODD and, therefore, has a triple-action effect.
5. Peripherally acting μ-opioid receptor antagonists (PAMORAs)
  Naldemedine, a PAMORA, is covered by insurance only for OIC in Japan [45]. OIC is caused by decreased water retention, decreased intestinal peristalsis, and contraction of the anal sphincter, and the mechanism of action of naldemedine suggests that it can ameliorate these effects. Until now, OIC has mainly been treated using MgO; however, in a randomized controlled trial in which either MgO or naldemedine was administered simultaneously with opioid initiation as a prophylactic treatment for OIC, naldemedine showed no worsening of the Japanese version of the Patient Assessment of Constipation Quality of Life (JPAC-QOL) score after 2 weeks compared with MgO; additionally, complete spontaneous bowel movements were significantly higher, and the incidence of nausea was significantly lower [46]. We have also investigated the changes in defecation frequency and quality of life after administering various laxatives to patients with OIC to determine which laxatives are effective in treating OIC. The results showed no difference in defecation frequency among conventional and novel laxatives and naldemedine. However, compared with conventional laxatives, naldemedine and the novel laxatives elobixibat and lubiprostone significantly improved defecation-related quality of life; additionally, naldemedine and elobixibat improved defecation-related symptoms. Thus, the prevention and treatment of OIC are expected to transition towards naldemedine. According to recent Japanese guidelines, if symptoms of constipation are observed, the first priority is to deal with drug-induced constipation, especially OIC. Therefore, if there is a possibility of OIC, naldemedine is recommended [15]; notably, it can be used simultaneously with both weak and strong opioids [26].

## 3. Recommended Care and Treatments Based on Rectal Constipation Ultrasonography and Discussion

As depicted in the flowchart in Figure 1, ultrasonography can be used to classify rectal fecal impaction into three categories. Patients in all three categories should be guided and cared for in the least invasive manner possible before initiating drug therapy as and when required; however, all choices should be made according to the patient’s condition and the situation in the ward, outpatient clinic, or home setting. We believe that this flowchart can help ascertain the status of fecal impaction in the rectum using objective ultrasonography findings to provide the most optimum and appropriate care and treatments. Until now, care and drug therapy (e.g., oral medications, suppositories, and enemas) have been administered without considering ultrasonography findings. However, in the absence of information regarding stool retention status, these treatments are merely empirical and can result in suboptimal care and treatment. For example, suppositories or enemas may have been used to treat patients despite the absence of stool in the rectum, resulting in unnecessary discomfort to patients. Although rectal examination may be useful for determining the status of rectal stools, we believe that more systematic and evidence-based treatment is possible by devising appropriate strategies based on objective ultrasonography indices, especially since this involves a less invasive procedure and minimizes patient discomfort.

### 3.1. No Fecal Retention in the Rectum

Action required: transportation of stools to the rectum.

Recommended guidance: lifestyle modification, diet.

Recommended care: care to promote intestinal peristalsis.

Recommended drug therapy:

1. Ileal bile acid transporter inhibitor (elobixibat, [Goofice^®^]).
2. Stimulant laxative (Sodium picosulfate^®^), rescue use only.

As shown in Figure 1, if the findings indicate that there is no rectal stool retention, lifestyle modifications related to diet, exercise, and sleep and dietary therapy should be implemented. However, if patients have difficulty communicating, this aspect should be given due consideration. From the nursing perspective, care that promotes intestinal peristalsis should be selected. If effective, continue the treatment; if the stools are hard (Bristol scale 1 to 2), select an osmotic laxative (MgO or polyethylene glycol). In such cases, priority should be given to polyethylene glycol for older patients and patients with renal dysfunction and those using MgO for more than 1 month or more than 1650 mg MgO, as these patients are at risk for hypermagnesemia. In addition, if the patient has undergone total gastrectomy or is using acid secretion inhibitors, the effect of MgO is reduced, and polyethylene glycol should be preferred in such cases as well. If lifestyle modification and dietary therapy are ineffective, priority should be given to IBAT inhibitors (Goofice^®^, EA pharma, Tokyo, Japan), as they require hyperperistalsis to transport stools to the rectum, and basic and clinical data have confirmed that bile acids induce hyperperistalsis [47]. On the other hand, osmotic laxatives, especially polyethylene glycol, have a first bowel movement time comparable to that of placebo and are not expected to promote colonic fecal transport [32]. Although intestinal secretagogue agents, mainly lubiprostone, have been reported to improve small intestinal transit time [48], their effect on colonic motility is not significant, and they do not promote colonic peristalsis [15]. 

Abortive use of a stimulant laxative should be the second-line drug therapy. Because of the potential for abuse, dependence, and colonic inertia with the use of stimulant laxatives, abortive use is recommended only one to two times per week [15]. 

In summary, an IBAT inhibitor or stimulant laxative should be the first drug to be administered. If a stimulant laxative is used, caution should be exercised to ensure that there is no bowel obstruction or any condition requiring urgent attention prior to administration. If this approach is ineffective, the patient should be re-evaluated after consultations with specialists, including proctologists, or ultrasound. Ward nurses and nurses in the home setting should report to the attending physician and responsible personnel. 

### 3.2. Fecal Retention in the Rectum, but No Hard Fecal Impaction 

Action required: creating a toilet-going environment and stimulating more bowel movements.

Recommended instruction: guide the patient to promote using the toilet.

Recommended care: care to stimulate stool mass evacuation and intestinal peristalsis.

Recommended treatment:

1. Suppository (sodium bicarbonate/anhydrous sodium dihydrogen phosphate suppository [New lecicarbon^®^]).
2. Osmotic laxatives (MgO or PEG [Movicol^®^]).
3. IBAT inhibitor (elobixibat [Goofice^®^]) [preferred, especially in cases of LODD].

These findings indicate that the soft stools have reached the rectum. Therefore, the patient should first be guided to use the toilet and encouraged to defecate independently. The next recommended care is to promote stool mass evacuation and intestinal peristalsis. If this is effective, it should be continued; if hard stools (Bristol Scale 1 or 2) are observed, osmotic laxatives should be used. 

If this guidance and care prove ineffective, suppositories should be used first. In particular, lecicarbon suppositories are considered optimal because they produce carbon dioxide and promote rectal peristalsis. Next, osmotic laxatives and bile acid reuptake inhibitors should be used. Bile acid reuptake inhibitors improve bowel movements and should be preferred, especially for patients who have lost bowel movement.

### 3.3. Hard Fecal Retention in the Rectum 

Action required: inducing the excretion of hard stools and normal defecation.

Recommended instruction: promote self-defecation when possible.

Recommended care: fecal disimpaction.

Recommended treatment: enema because of stool evacuation after manual maneuvers.

Maintenance treatment after defecation:

1. IBAT inhibitor (elobixibat [Goofice^®^]) [preferred, especially in cases of LODD].
2. Osmotic laxatives (polyethylene glycol [Movicol^®^]).

If a patient is found to have hard stool retention, the first step is to encourage self-defecation. If this is difficult, it is important to manually remove as much hard stool as possible by excreting it first to create space for fluid to enter between the enema and the rectal wall. If an enema is given without stool evacuation, there is a possibility that the enema liquid may not enter or enter the hard stools and thus be ineffective. The Japan Nurses Association and PMDA have reported complications such as rectal perforation and hemolysis due to inappropriate enemas and have issued a warning about enemas [49]. Therefore, slow injection in the left lateral recumbent position is important to avoid contact with the anterior rectal wall. Maintenance therapy is often required after the evacuation of hard stools by evacuation and enema. IBAT inhibitors have been reported to significantly reduce evacuation and enemas. Polyethylene glycol has been reported to be effective against fecal impaction [50,51]. Therefore, the use of IBAT inhibitors or polyethylene glycol as a maintenance therapy is recommended. However, in patients with loss of bowel movement, IBAT inhibitors should be preferred. If the patient is refractory to drug therapy, the patient should be re-evaluated, or a specialist, such as a proctologist should be consulted. 

## 4. Discussion

This study is the first in the world to develop a care and treatment algorithm based on rectal echocardiographic findings for patients with constipation, based on a review of the existing literature and expert opinions from a multidisciplinary team. However, the optimal care and treatment of patients after evaluation is not yet possible [13,14,15]. However, there are no reports on optimal care and treatment after evaluation. To fill this research gap, we developed a care and treatment algorithm based on a review of the existing literature and expert opinion.

Chronic constipation is classified into two types: decreased frequency of defecation and difficult defecation [15]. The decreased defecation frequency type is further classified into normal and slow transit constipation types by measuring the colonic transit time [15]. According to the latest Japanese guidelines, abdominal ultrasonography is useful in diagnosing patients with obstructed bowel movements or those with stool retention in the rectum and symptoms of defecation difficulty (straining, a feeling of incomplete evacuation, obstructive sensation) [15]. Suppose the patient is of the obstructed defecation type. In that case, the priority is transanal treatment such as suppositories, enemas, and other topical medications. If the patient has a decreased defecation frequency type, the treatment choice is oral constipation medications [15]. Therefore, visualization of fecal retention in the rectum by abdominal ultrasonography may enable evidence-based treatment with a high degree of satisfaction and may also have an impact on medical economics by reducing unnecessary fecal disimpassion and external procedures such as suppositories and enemas. Therefore, we believe that the care and treatment algorithm based on ultrasonographic findings developed in this study can be used to achieve a high level of patient satisfaction and to take healthcare economics into consideration.

Regarding the economics and feasibility of this consensus document, we believe it is economical because it can eliminate unnecessary enemas and other trans-anal procedures and reduce complications (gastrointestinal perforation and hemolysis) caused by unnecessary trans-anal procedures. In addition, it is highly feasible because handheld ultrasonography has been introduced to home and nursing care facilities, as well as stand-type ultrasonography in many hospitals and clinics, and transanal procedures (disimpaction, suppositories, and enemas) and oral constipation medications are widely used not only in hospitals but also in home and nursing care facilities.

Limitations of this study are (1) it is an expert opinion in this field consisting of multiple professions, but it is not a survey of non-specialists, (2) only Japanese, and (3) care and treatment were not provided to the patients. In other words, there is no validation. Therefore, as a future Challenge, it is important to use the treatment algorithm based on the ultrasonographic findings constructed in this study and validate it by using it on patients in the real world. Future work is needed to conduct validation.

## 5. Conclusions

Existing evidence and expert opinions suggest that the care and treatment of patients with constipation can be based on rectal ultrasonographic findings. Nonetheless, future validation of care and treatment strategies based on this flowchart is recommended.

## Figures and Tables

**Table 1:** Characteristics of various constipation medications a table.

Classification	Sub-classification	Common name	Product Name	Form	Compliance (times/day)	Administration timing	Dosage	Adaptation	Mechanism of action	Water secretion action	Peristaltic stimulant action	Recovery of defecation desire	Low nursing and caregiving burden	Contraindications	Notes
Bulking agents	□	Polycarbophil calcium	Coronel^®^ Porifuru^®^	Tablet Powder	1–3	After meals	1.5–3.0 g/day	Stool abnormality (diarrhea/constipation) and gastrointestinal symptoms in irritable bowel syndrome	Include large amounts of water in stools	○	□	□	□	Acute abdomen Postoperative ileus Hypercalcemia Renal failure, renal stones	□
		Carmellose sodium	Carmellose sodium	Powder	1–3	After meals	1.5–6.0 g/day Adjustable according to age and symptoms.	Constipation	Absorbs water in the intestine, expands to form a gel, increases the volume of the fecal mass, and physically stimulates the intestinal wall.	○	□	□	□	Acute abdomen Severe indurated stool	□
Osmotic laxatives Note: Select polyethylene glycol in cases of elderly patients, renal dysfunction, patients on acid secretion inhibitors, and a prior total gastrectomy procedure	Sugar-based laxative	Lactulose	Monilak^®^	Syrup	1–3	After meals	Adult: 19.5–39.0 g/day Children: 0.33–1.30 mg/kg Adjustable according to age and symptoms.	Constipation (limited to children), Chronic constipation (except organic disease), stool abnormality after obstetric/gynecologic surgery	It reaches the large intestine without being degraded or absorbed in the small intestine, and the osmotic effect of the lactulose unchanged form promotes the movement of water into the intestine.	○	□	□	○	Patients with lactose/galactosemia	□
			Ragnos NF^®^	Jelly	1–3		Two packets at a time (max. 6 packets) Adjustable according to age and symptoms.								
	Salt-based laxatives	Magnesium oxide	Magnesium oxide	Tablet Powder	1–3	After meals Before bedtime	2 g/day Adjustable according to age and symptoms.	Constipation, gastric/duodenal ulcer Abnormal upper gastrointestinal function	The osmotic pressure of magnesium salts promotes water transfer into the intestinal tract	○	□	□	○	□	Be aware of hypermagnesemia # (especially in cases of impaired renal function). Some deaths have occurred. Prone to causing hypermagnesemia with concomitant use of active vitamin D preparations. Many concomitant medications * should be used with caution. Weak effect if acid secretion inhibitors are taken orally or after total gastrectomy.
	□	Polyethylene glycol	Movicol^®^	Powder	1–3	After meals Before bedtime	Infants 2 to 7 years: 6.85 g/day –27.4 g/day Children 7 to 12 years: 13.7 g/day –27.4 g/day Children over 12 years and adults: 13.7 g/day –41.1 g/day Adjustable according to age and symptoms.	Chronic constipation (excluding organic diseases)	The osmotic pressure of polyethylene glycol promotes water transfer into the intestinal tract	○	□	□	□	Intestinal obstruction, intestinal perforation, severe inflammatory bowel disease	Less dependent and addictive. Large amount of powder. No pills. No contraindications or precautions.
	Contact laxative	Casanthranol/Dioctyl sodium sulfosuccinate	Vemas^®^ combination tablet	Tablet	1–3	After meals Before bedtime	2–3 tablets once or 5–6 tablets once before bedtime Adjustable according to age and symptoms	constipation, elimination of intestinal contents during abdominal organ examination or before and after surgery	Surfactant action lowers the surface tension of stools, making them moist and soft	○	○	□	□	Acute abdomen, severely indurated stool	Yellowish brown or red urine
Intestinal secretagogues	Chloride channel activator	Lubiprostone	Amitiza^®^	Capsule	1–2	After meals	12–48 μg/day Can be adjusted according to age and symptoms	Chronic constipation (excluding organic diseases)	Activates ClC-2 chloride ion channels, thereby promoting water secretion into the intestinal tract	○	□	□	○	Intestinal obstruction, pregnancy	Nausea is common in young women.
	Guanylate cyclase C receptor agonists	Linaclotide	Linzess^®^	Tablet	1	Before meals	0.25–0.5 mg/day Adjustable according to age and symptoms	Chronic constipation (excluding organic diseases), irritable bowel syndrome constipation type	Acts on guanylate cyclase C receptors on the surface of intestinal epithelial cells to promote water secretion into the intestinal tract	○	□	□	□	Intestinal obstruction	Effective for abdominal pain in irritable bowel syndrome. No contraindications or precautions related to concomitant medications.
Ileal bile acid transporter inhibitors	□	Elobixibat	Goofice^®^	Tablet	1	Before meals	5–15 mg/day Adjustable according to age and symptoms	Chronic constipation (excluding organic diseases)	Inhibits reabsorption of bile acids in the ileum	○	○	○	□	Intestinal obstruction	Effective for both water transfer and peristalsis promotion. Effective in improving bowel movements.
Small bowel stimulant laxatives	□	Castor oil	Castor oil	Liquid	□	On demand	Adults: 15–30 mL per dose (maximum: 60 mL), Children: 5 to 15 mL per dose, Infants: 1 to 5 mL per dose, Adjustable according to age and symptoms.	Constipation, elimination of intestinal contents before and after surgery	It is broken down into glycerol and ricinoleic acid by the action of lipases in the small intestine. Ricinoleic acid stimulates the small intestine to stimulate defecation.	□	○	□	□	Acute abdomen, severely indurated stool	□
Large intestine stimulant laxatives Note: For rescue use only.	Anthraquinone	Senna/Sennoside	Plusenide^®^ Sennoside^®^	Tablet	1	Before bedtime	12–48 mg/times Adjustable according to age and symptoms	Constipation	Improves peristalsis of the large intestine by producing rainanthrone through the action of intestinal bacteria	□	○	□	○	Acute abdomen, severely indurated stools, electrolyte imbalance (especially hypokalemia)	Colonic melanosis. Dependent and addictive. Yellowish brown or red urine.
			Alosenn^®^	Powder	1–2	Before meals After meals	0.5–1.0 g/ times Adjustable according to age and symptoms	□	□	□	□	□	□	□	□
	Diphenyl	Sodium picosulfate	Laxoberon^®^ Sodium picosulfate^®^	Liquid Tablet	1	After meals Before bedtime	Adults: 2.5 mg–7.5 mg (10–15 drops) 7–15 years: 5 mg (10 drops) 4–6 years: 3.5 mg (7 drops) 1–3 years: 3 mg (6 drops) 7–12 months: 1.5 mg (3 drops) 6 months and younger: 1 mg (2 drops) Adjustable according to age and symptoms	Constipation, postoperative defecation aid, elimination of intestinal contents prior to colonoscopy (radiography and endoscopy) and surgery	Diphenyl bodies generated by allylsulfatase derived from intestinal bacteria stimulate the colonic mucosa and inhibit water absorption in the large intestine	□	○	□	○	Acute abdomen	No colonic melanosis. Less addictive. Acceptable for young children, pregnant women, and the elderly.
External agents/procedures	Suppository	Bisacodyl suppository	Teleminsoft^®^	Suppository	1–2	On demand	1–2 per day Adjustable according to age and symptoms	Constipation, elimination of intestinal contents during gastrointestinal examination or before and after surgery	Selectively acts on the colon and rectal mucosa to promote peristalsis	□	○	□	□	Acute abdomen	□
		Sodium□bicarbonate/anhydrous sodium□dihydrogen phosphate suppository	New lecicarbon^®^	Suppository	On demand	On demand	1–3 per day	Constipation	Produces carbon dioxide gas, which stimulates the rectal mucosa and dilates the rectum to promote defecation through the diastolic reflex	□	○	□	□	□	Can be used for pregnant and parturient women.
	Enema	Glycerin enema	Glycerin	Enema	On demand	On demand	30–60 mL per application	Constipation, defecation during bowel disease	The concentrated liquid stimulates the intestines and promotes bowel movement. It also penetrates the stool and softens it	○	○	□	□	Intra-intestinal hemorrhage, intra-abdominal inflammation, intestinal perforation	Urine color (hemolysis)
Peripherally acting μ-opioid receptor antagonist	□	Naldemedine	Symproic^®^	Tablet	1	After meals	0.2 mg/day	Opioid-induced constipation	Antagonizes intestinal μ-opioid receptors	○	○	□	○	Gastrointestinal obstruction	□

# Symptoms of hypermagnesemia: nausea, vomiting, dry mouth, hypotension, bradycardia, skin flushing, muscle weakness, somnolence, etc. * Decreases the effectiveness of the following drugs: tetracyclines, new quinolones, bisphosphonates, celecoxib, rosuvastatin, rabeprazole, gabapentin, calcium polycarbophil, ion-exchange resins for improving hyperkalemia. ○ mark indicates ‘Yes’.

## Data Availability

Data are contained within the article.
